# An epidemic of spastic paraparesis of unknown aetiology in Northern Mozambique

**DOI:** 10.11604/pamj.supp.2017.27.1.12623

**Published:** 2017-05-28

**Authors:** Cátia Luciana Abdulfattáhe Taibo, Julie Cliff, Hans Rosling, Casey Daniel Hall, Meeyoung Mattie Park, Joseph Asamoah Frimpong

**Affiliations:** 1 Microbiology Department, Eduardo Mondlane University, Maputo, Mozambique; 2 Mozambique Field Epidemiology and Laboratory Training Program, Mozambique; 3 Ministry of Health, Maputo, Mozambique; 4 Nacala District Health Department, Nacala, Mozambique; 5 Rollins School of Public Health, Emory University, Atlanta, USA; 6 Liberia Field Epidemiology Training Program, Liberia

**Keywords:** Public health, epidemiology, spastic paraparesis, epidemic, outbreak investigation

## Abstract

This case study is based on a real-life outbreak investigation undertaken in Mozambique in 1981. This case study describes and promotes one particular approach to unknown disease outbreak investigation. Investigational procedures, however, may vary depending on location and outbreak. It is anticipated that the epidemiologist investigating an unknown disease outbreak will work within the framework of a “multidisciplinary investigation team”. It is through the collaborative efforts of this team, with each member playing a critical role, that outbreak investigations are successfully completed. Some aspects of the original outbreak and investigation have, however, been altered to assist in meeting the desired teaching objectives and to allow completion of the case study in less than 3 hours.

## How to use this case study

**General instructions:** Case studies in applied epidemiology allow students to practice applying epidemiologic skills in the classroom to address real-world public health problems. The case studies are used as a vital component of an applied epidemiology curriculum, rather than as stand-alone tools. They are ideally suited to reinforcing principles and skills already covered in a lecture or in background reading. Ideally, 1 or 2 instructors facilitate the case study for 8 to 20 students in a classroom or conference room. The instructor directs a participant to read aloud a paragraph or two, going around the room and giving each participant a chance to read. When the participant reads a question, the instructor directs all participants to perform calculations, construct graphs, or engage in a discussion of the answer. Sometimes, the instructor can split the class to play different roles or take different sides in answering the question. As a result, participants learn from each other, not just from the instructors. Additional instructor’s notes are included with each question. The instructors must keep Part 4 of the student guide (last 6 pages) as a separate handout. Do not let students see Part 4 until after they have completed Part 3.

**Audience:** residents in Field Epidemiology Training Programs (FETPs), Field Epidemiology and Laboratory Training Programs (FELTPs), Epidemic Intelligence Service (EIS) programs and others who will be engaged in conducting field studies involving humans, or who are interested in this topic.

**Prerequisites:** before using this case study, participants should have working knowledge of descriptive epidemiology, epidemic curves, measures of association, study design, and outbreak investigation. The student will also benefit from having some familiarity with paralysis of unknown aetiology, or unknown disease outbreaks investigation techniques, but will be likely to rely heavily on others with greater expertise in these areas in a real-life outbreak situation.

**Materials needed:** a calculator, the case control study student guide

**Level of training and associated public health activity:** basic to intermediate, i.e., this case study could be used in an introductory course in field epidemiology – outbreak investigation

**Time required:** approximately 3 hours

**Language:** English

## Case study material

- Download the case study student guide (PDF - 1.11 MB)
- Request the case study facilitator guide
